# Improving the Performance of Anthropometry Measurements in the Pediatric Intensive Care Unit

**DOI:** 10.1097/pq9.0000000000000022

**Published:** 2017-05-10

**Authors:** Vijay Srinivasan, Stephanie Seiple, Monica Nagle, Shiela Falk, Sherri Kubis, Henry M. Lee, Martha Sisko, Maria Mascarenhas, Sharon Y. Irving

**Affiliations:** From the * Department of Anesthesiology and Critical Care Medicine, Children’s Hospital of Philadelphia, Philadelphia, Pa.; † Department of Anesthesiology and Critical Care, Perelman School of Medicine at the University of Pennsylvania, Philadelphia, Pa.; ‡ Department of Clinical Nutrition, Children’s Hospital of Philadelphia, Philadelphia, Pa.; § Department of Nursing, Children’s Hospital of Philadelphia, Philadelphia, Pa.; ¶ Davidson College, Davidson, N.C.; ‖ Division of Gastroenterology, Hepatology and Nutrition, Department of Pediatrics, Children’s Hospital of Philadelphia, Philadelphia, Pa.; and ** Department of Family and Community Health, University of Pennsylvania School of Nursing, Philadelphia, Pa.

## Abstract

**Introduction:**

Obtaining anthropometry measurements in critically ill children is challenging. Our objective was to improve the process of obtaining anthropometry measurements in the pediatric intensive care unit (PICU; even if previously obtained) using a dedicated PICU nutrition support team (NST).

**Methods:**

PICU staff were trained to perform anthropometry measurements through online education, skills training, and just-in-time bedside teaching by the PICU NST. Equipment was upgraded and standardized throughout the PICU along with implementation of preselected orders in the electronic medical record. Data were collected before and immediately after intervention and at monthly intervals from 12 to 36 months to test sustainability of practice change. PICU staff were surveyed on barriers to anthropometry measurements at 36 months after initial intervention.

**Results:**

Compared with baseline, the intervention resulted in more patients with orders for weight, stature, and head circumference (all *P* < 0.001) at PICU admission. Correspondingly, more patients had measurements of weight (*P* = 0.04), stature (*P* = 0.01), and head circumference (*P* = 0.009) at PICU admission. For long-stay patients (>7 days), compliance improved with measurements of serial weights (*P* = 0.002), stature (*P* < 0.001), and head circumference (*P* = 0.02). Between 12 and 36 months after the intervention, there was a noticeable trend to increases in weight measurements at PICU admission, and to a lesser extent, of stature and head circumference. Competing clinical priorities were a key barrier to anthropometry measurements.

**Conclusions:**

Performance of anthropometry measurements in the PICU can be improved by a dedicated PICU NST; however, sustaining these improvements is challenging due to competing clinical priorities.

## Abstract 2

Supplemental Digital Content is available in the text.

## INTRODUCTION

Malnutrition is a major risk factor for adverse outcomes in critically ill children with associated higher rates of readmission and health-care costs.^1^ Current estimates of the prevalence of malnutrition in critically ill children vary widely from 6 to 51% depending on patient diagnoses and criteria used to identify malnutrition.^2–4^ In 1995, the Joint Commission mandated assessment of nutritional status in hospitalized patients within 24 hours of admission.^5^ Age-appropriate measurements of anthropometry consisting of weight, stature, and head circumference constitute an important part of nutritional status assessment and remain vital for the safe and effective care of critically ill children in the pediatric intensive care unit (PICU).^6^ In addition to assessment of nutritional status, anthropometry measurements are also vital to calculate the correct dosage of medications and blood products, prescribe nutrient intake, and determine appropriate therapies and equipment needs for critically ill children.^7,8^ Serial anthropometry measurements are crucial to understand fluctuations in fluid balance and changes in body mass during the course of critical illness. Such serial measurements assume even greater importance in neonates, infants, and young children who also experience ongoing growth during and after their recovery from critical illness.^9^

There are numerous challenges to obtaining anthropometry measurements in critically ill children due to severity of illness and associated necessary technological interventions. The importance of these measurements and their impact on patient care may not be appreciated by clinicians when faced with competing priorities related to airway management, ventilation support, and hemodynamic therapies that invariably take precedence in a critically ill child. Consequently, anthropometry measurements may be inconsistently obtained, completely avoided, or estimated as a matter of routine practice. Weight, arguably the most important anthropometric measurement, is frequently documented based on “best-guess” estimates by the clinical care team or parent/caregivers, with varying degrees of accuracy.^10–12^ Stature and head circumference are frequently not performed due to perceived lack of importance while caring for a critically ill child. Inaccurate and inconsistent anthropometry measurements can thus pose significant risks to the management of a critically ill child, with great potential for harm.

We had previously conducted a multiinstitutional international survey of PICU staff perceptions of barriers to obtaining anthropometry measurements in the PICU.^13^ In addition to identifying barriers, we also observed that perceptions of these barriers to anthropometry measurements differed widely between ordering providers and nurses. Following the results of this survey, we sought to overcome these gaps and barriers to anthropometry measurements in our PICU through targeted quality improvement (QI) methods. The objective of this project was to improve and sustain the process of obtaining anthropometry measurements in critically ill children admitted to the PICU using QI methods implemented by a dedicated PICU nutrition support team (NST).

## METHODS

### Clinical Setting

The PICU NST undertook this QI project between December 2012 and September 2016 to improve the process of obtaining anthropometry measurements in the 55 bed multidisciplinary noncardiac PICU at The Children’s Hospital of Philadelphia. Our PICU standards (and hospital standards) require that all age-appropriate anthropometry measurements be completed within 24 hours of admission to the PICU, even if previously obtained before admission to the PICU. The typical workflow consists of the ordering provider (physician or designee) placing orders in the electronic medical record (EMR) to obtain anthropometry followed by the bedside registered nurse (RN) completing the anthropometry measurements with appropriate documentation in the EMR. Our multidisciplinary PICU NST consists of PICU-specific registered dietitians (RDs), pediatric critical care advanced practice nurses, pediatric critical care RNs, a pediatric critical care pharmacist, a pediatric gastroenterologist (board certified in nutrition), and a pediatric intensivist. This QI project was deemed exempt by the The Children’s Hospital of Philadelphia Institutional Review Board.

### QI Interventions

**Fig. 1.**
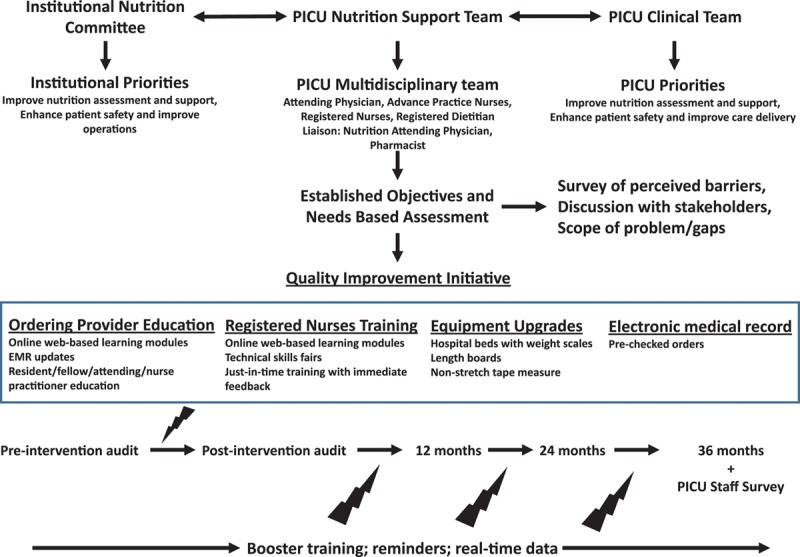
Summary of QI interventions with PICU NST.

The QI methods developed by the PICU NST were targeted to ordering providers as well as nurses and were implemented over a 3-month period by the PICU NST. These interventions included Web-based online education utilizing Power Point slides to review the importance of anthropometry measurements and detailed descriptions of the correct age-appropriate techniques to obtain such measurements. Additional hands-on training was provided at clinical practice skills fairs to reinforce PICU RN education. The online training and the skills fair education were mandatory components of the quarterly instruction for PICU RNs. A third layer of bedside, “just-in-time” training was provided to PICU RNs and nursing aides by the PICU NST within 12 hours of the child being admitted to the PICU. The “just-in-time” training methods used standardized scripts with the PICU-specific RDs supervising training of RNs and nursing aides at the bedside with use of actual equipment to demonstrate the correct approach to obtaining anthropometry measurements in a consistent manner. Equipments to perform anthropometry were upgraded and standardized throughout the PICU and stored in standard locations to facilitate performance of anthropometry measurements. The equipment upgrades included beds capable of electronically weighing patients and length boards. The time and date of obtaining the most recent weight and the actual weight value were added to the PICU RN bedside rounding script as a prompt to discuss the importance of weight measurement during daily bedside rounds. Lastly, orders to obtain anthropometry measurements were preselected in the PICU admission order set in the EMR. Figure 1 summarizes the QI methods employed in this project. Training and education reminders were sent to ordering providers and nurses at regular intervals by the PICU NST during this QI project via group e-mail distribution lists and the use of screen savers on desktop computer workstations. Additional methods used for staff education and reminders included notices posted on staff bulletin boards and regular announcements with real-time implementation updates in the recurring PICU multidisciplinary QI meetings.

### Data Collection

Baseline (preintervention) data on anthropometry orders and measurements following PICU admission were collected by the PICU RDs at weekly intervals over a 3-month period prior to institution of QI methods. The PICU RDs performed manual audits of the EMR of all children present in the PICU on the specific day of chart audit. There was no prior notice given to clinical providers, including nursing staff, of their intent. Data collection on any given day was determined by PICU RD availability, and any day in which the RD could not complete data collection was not included in the analysis. Postintervention data were similarly collected by the PICU RDs at weekly intervals over a 3-month period via manual audits to ensure consistency with baseline preintervention data collection. The PICU RDs did not provide any prior notice of their intent to clinical staff. Data collection on any given day was determined by PICU RD availability, and any day in which the RD could not complete data collection was not included in the analysis. Further follow-up data collections were done at monthly intervals from 12 to 36 months using electronic data capture from the EMR to test sustainability of practice change. Additionally, the PICU staff were surveyed at the end of 36 months to assess ongoing barriers to anthropometry measurements in the PICU and solutions for improvement.

### Ongoing Education and Interventions

Following the initial QI intervention, the PICU NST continued to share details of performance data at monthly intervals at divisional QI meetings. These details were in the form of graphical trend data with a required minimum standard of completed anthropometry measurements in at least 80% of PICU patients for the respective time periods. Summary results were distributed via e-mail to all PICU staff to highlight deficiencies. Information flyers on staff bulletin boards and screensavers on computer workstations were utilized to emphasize the rationale for and importance of anthropometry measurements as well as to share key results of the QI initiative to sustain frontline clinicians’ interest and involvement. Accountability to meet target expectations for anthropometry measurement was jointly shared between the PICU NST and PICU nursing leadership and reviewed at regular intervals.

### Study Outcomes

The primary outcome was changes in frequency of anthropometry measurement orders placed and actual measurements obtained at PICU admission. Secondary outcomes were compliance of measurements with orders in long-stay PICU patients (length of stay > 7 days). Patient-specific characteristics as well as PICU-related interventions were analyzed with regard to anthropometry measurements. Balancing metrics included time spent by bedside RNs to carry out measurements as well as time involvement of the PICU NST to facilitate these efforts. Staff perceptions of ongoing barriers to anthropometry measurements and solutions to overcome these barriers were also examined.

### Statistical Analysis

Descriptive data were summarized as medians with interquartile ranges for data that were not normally distributed and as counts with percentages for categorical data. Wilcoxon rank-sum test was used to compare differences between preintervention and postintervention groups for continuous variables that were not normally distributed. Fischer’s exact test or chi-square test were used to analyze differences in categorical variables across preintervention and postintervention groups. Graphical trend data were represented in the form of control charts. Data were analyzed using Stata 14 (StataCorp, College Station, Tex.). A *P* value less than or equal to 0.05 was considered significant for all analyses. Comparisons between preintervention and postintervention time points were not adjusted for multiple comparisons and were treated equally.

## RESULTS

The preintervention observation period provided complete data on 357 subjects. Following institution of targeted QI methods with staff training approaching 95%, the postintervention observation period yielded complete data on 296 subjects. Both preintervention and postintervention cohorts were comparable with each other with regard to demographic and admission characteristics, as well as key PICU interventions and outcomes.

Compared with the preintervention period, more patients in the postintervention period had orders placed in the EMR for weight (52–82%; *P* < 0.001), stature (4–85%; *P* < 0.001), and head circumference (10–33%; *P* < 0.001) at PICU admission (Table 1). Correspondingly, more patients in the postintervention period had completed measurements of weight (64–72%; *P* = 0.04), stature (27–36%; *P* = 0.01), and head circumference (7–19%; *P* = 0.009) at PICU admission. For long-stay PICU patients, there was improved compliance of measurements with orders for measurements of serial weights (23–40%; *P* = 0.002), stature (5–32%; *P* < 0.001), and head circumference (7–16%; *P* = 0.02; Table 2).

**Table 1.**
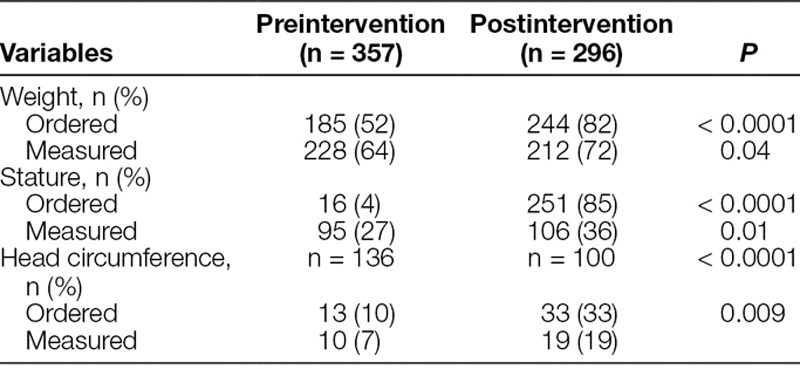
Anthropometry Orders and Measurements at Admission in Preintervention and Postintervention PICU Cohorts

**Table 2.**
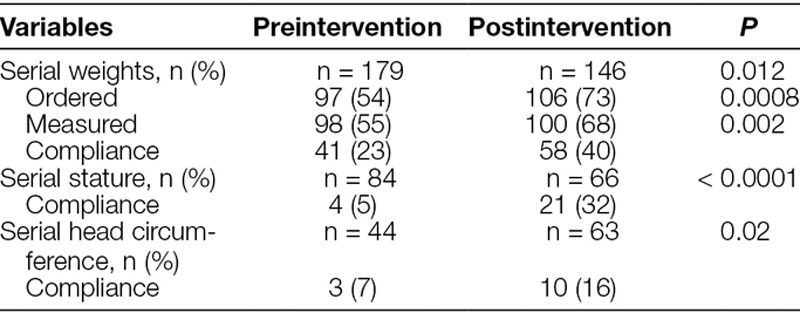
Compliance of Anthropometry Orders with Measurements during Admission: Long-Stay Patients (> 7 Days) in Preintervention and Postintervention PICU Cohorts

Changes in weight and stature measurements by patient-specific characteristics as well as PICU-related interventions from the preintervention period to the postintervention period are depicted in Table 3. Compared with the preintervention period, there were substantial increases in weight and stature measurements during the postintervention period for patients who were electively admitted to the PICU, postoperative, admitted on weekdays, admitted between 2:00 and 10:00 pm, with either a central venous catheter or arterial catheter, and requiring invasive mechanical ventilation. In addition, patients requiring vasoactive infusions and those with an oncologic diagnosis also experienced a significant increase in stature measurements.

**Table 3.**
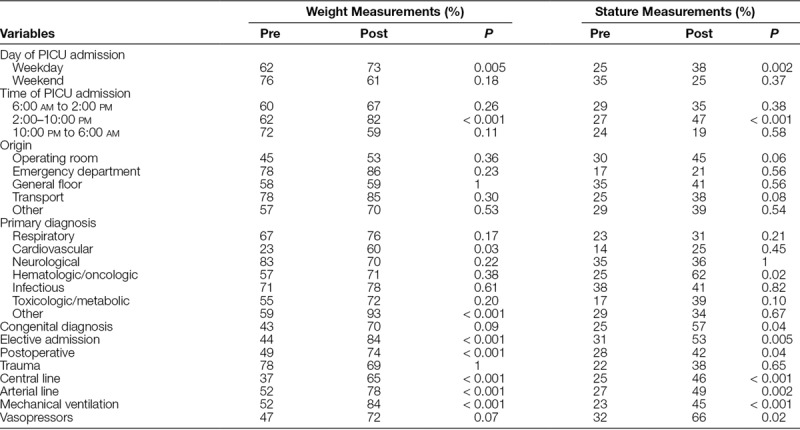
Changes in Weight and Stature Measurements: Patient-Specific and PICU-Related Variables

**Fig. 2.**
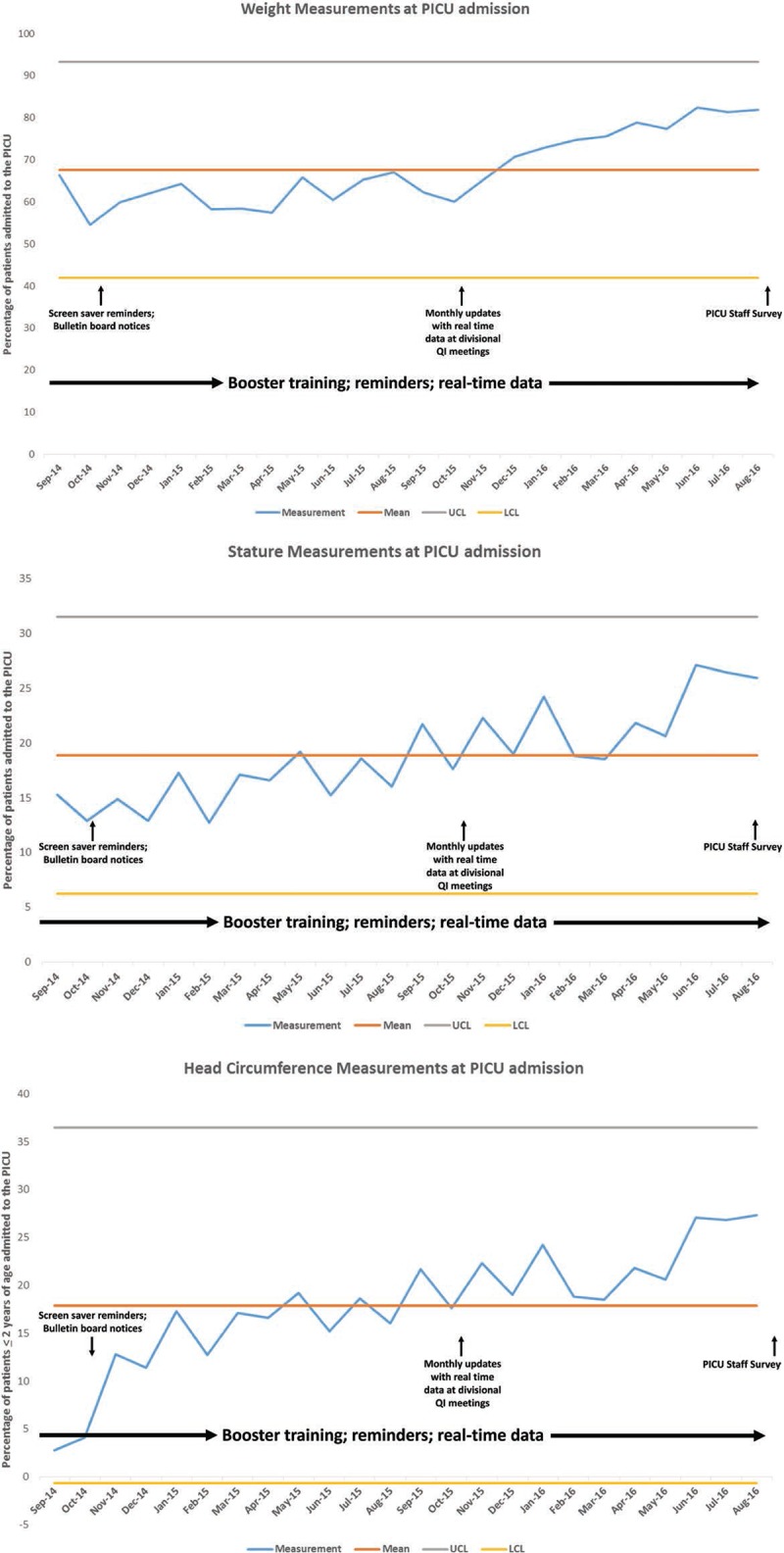
Control chart depicting trends in weight (A), stature (B), and head circumference measurements (C) at PICU admission. LCL, lower confidence limit; UCL, upper confidence limit.

Over a time period from 12 to 36 months after the intervention, there was a gradual and sustained trend with increases in weight measurements at PICU admission compared with the preintervention period. To a lesser extent, there were trends in increases of stature and head circumference measurements. The sustained increases in weight measurements at PICU admission were also noted in patients who were mechanically ventilated but not in those who were on vasoactive infusions. Graphical trends in measurements of weight, stature, and head circumference over the time period from 12 to 36 months are depicted in Figure 2A–C.

Our balancing metrics included time spent by the bedside RNs to obtain anthropometry measurements as well as time spent by the PICU NST on initial QI interventions as well as ongoing efforts to sustain these processes. Based on approximately 3,600 PICU admissions per year, bedside RN time for completion of measurements of weight, stature, and head circumference at PICU admission ranged from 1 to 2 full-time equivalent per week (FTE/week, with 1 FTE = 40 hours), depending on patient habitus and/or complexity of technological support. PICU NST time to support and maintain these QI processes ranged from 0.25 to 0.5 FTE/week.

The survey of PICU staff carried out at 36 months after initiation of QI interventions had a response rate of 31%, and results are provided in Table 4. Approximately 30% of respondents were ordering providers, whereas 66% were bedside RNs. The most common barrier perceived by staff was competing clinical priorities that prevented staff from being able to perform anthropometry measurements. The majority of staff felt that the presence of additional personnel would help improve the timely performance of anthropometry measurements after admission to the PICU. More bedside RNs than ordering providers perceived lack of additional personnel as barrier to measurements (56% versus 24%; *P* = 0.008). In contrast, more ordering providers than bedside RNs perceived lack of knowledge regarding importance of anthropometry measurements as a key barrier to measurements (48% versus 8%; *P* < 0.001). More ordering providers than bedside RNs felt that staff education regarding importance of anthropometry in the PICU was a key solution to improve anthropometry measurements (57% versus 14%; *P* < 0.001).

**Table 4.**
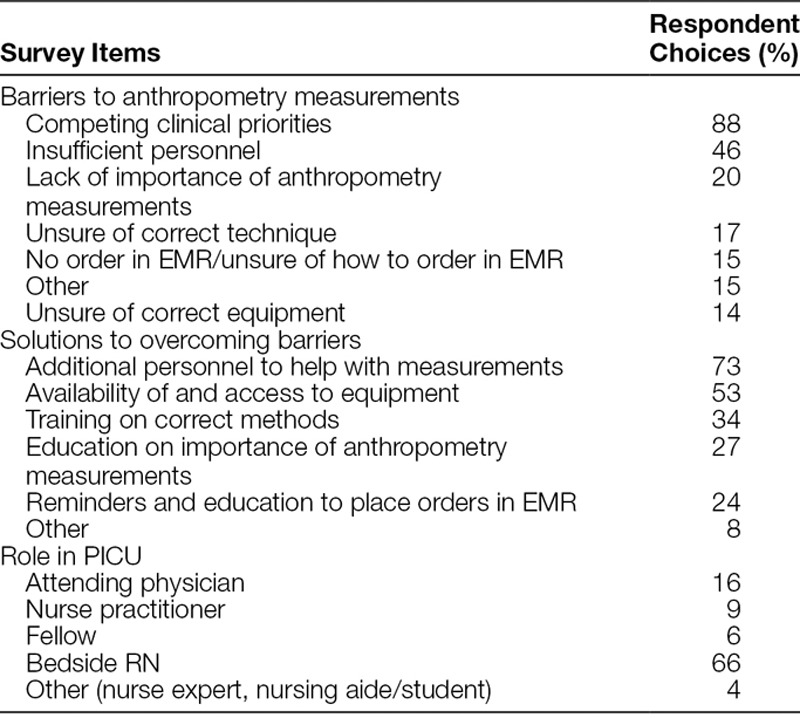
Staff Survey of Perceived Barriers to Anthropometry Measurements in the PICU: 36 Months after Initial QI Intervention

## DISCUSSION

The results of this project demonstrate that it is feasible to utilize targeted QI methods implemented by a dedicated PICU NST to improve the process of obtaining anthropometry measurements in the PICU. This improvement extends to sicker patients requiring extensive technological and hemodynamic support such as invasive mechanical ventilation, arterial and/or central venous catheters, and vasopressor infusions. We used a combination of targeted and well-studied QI methods such as Web-based online education, hands-on training at technical skills fairs and “just-in-time” training for the PICU clinical staff, in addition to upgrading and standardizing PICU equipment, adding prompts to the PICU RN bedside rounding script, and establishing preselected anthropometry orders in the PICU admission order set in the EMR. We observed that there was a sustained trend to improvements in weight measurements, and to a lesser extent, improvements in stature and head circumference measurements.

Nutritional status at admission to the PICU is strongly associated with both short-term and long-term outcomes from critical illness in children.^1–4^ Additionally, PICU-based interventions can influence serial changes in nutritional status. The optimal method(s) to measure nutritional status in the dynamic milieu of the critically ill child continues to be a subject of considerable debate among pediatric critical care providers worldwide.^6^ Measurement of anthropometric indices, particularly weight and stature, is a relatively easy method that is globally accepted and can be performed with accuracy and minimal training on readily available equipment. The advantages of using anthropometric indices largely stem from repeatability and comparability across different settings and regions. Major disadvantages include measurement errors (both intraprovider and interprovider), variability in equipment, lack of information pertaining to preillness anthropometry indices, and difficulty with accurately assessing changes in body mass in critical illness states due to fluid overload and/or dehydration.^14^

Previous studies have demonstrated significant shortfalls in obtaining anthropometry measurements in critically ill children and doing so accurately.^6,15,16^ Although actual weight measurements ranged from 42% to 88%, actual stature measurements ranged from 27% to 87% in these studies. This QI project builds on results from our multicenter, international survey of clinicians that examined perceived barriers to anthropometry measurements in the PICU^13^ (**see Table, Supplemental Digital Content 1**, http://links.lww.com/PQ9/A10). Our survey had demonstrated that most clinicians (84%) agree that obtaining anthropometry measurements on admission to the PICU is important, but few (3%) perceived that these were actually being done. Additionally, we observed significant differences in perception of barriers to anthropometry measurements based on provider role in the PICU. Based on the survey results, we targeted provider training and education as described in our QI methods. We also implemented preselected orders in the PICU EMR during this QI project to improve the process of obtaining anthropometry measurements. Interestingly, in the preintervention phase, we observed a higher frequency of completed measurements versus orders placed for weight (64% versus 52%; *P* < 0.05) and stature (27% versus 4%; *P* < 0.05). This suggests that the bedside RN providers are aware that these measurements were important for delivery of care in the PICU, even when not explicitly ordered to be obtained. PICU RN education and orientation typically emphasizes balancing the importance of obtaining these measurements with doing so safely in critically ill children who are frequently unstable and require multiple technological interventions; therefore, these results may be reflective of inherent nursing practice. However, in the postintervention phase, we observed that orders for anthropometry measurements did not reach 100% despite being preselected in the EMR PICU admission order set. This presents the intriguing possibility that ordering providers deselected the orders before signing off due to possible competing priorities and/or perceived lack of importance in the PICU setting.

We noted that the maximum improvements in anthropometry measurements occurred during weekdays between 2:00 and 10:00 pm, which likely reflects the availability of PICU RDs and additional staff during these timeframes to help with anthropometry measurements. Elective admissions and postoperative admissions to the PICU experienced a greater improvement in anthropometry measurements upon admission to the PICU, which may have been due to greater availability of staff and time to organize and implement patient care activities in a more planned manner. Importantly, the improvements also occurred in those PICU patients receiving extensive technological support such as invasive mechanical ventilation, arterial and central venous catheters, and vasopressor infusions, highlighting that it is feasible to complete anthropometry measurements even in perceived high-risk patients. Similar improvements were observed for compliance with orders for serial anthropometry measurements in long-stay PICU patients highlighting the feasibility to sustain this over the course of the PICU period of stay.

Although the trend in weight measurements demonstrated a noticeable improvement by 36 months to exceed 80% of patients admitted to the PICU, this was not the case with improvements in stature measurements. Even though equipment to measure stature was upgraded and standardized as part of this QI initiative, measurements of stature only increased up to a maximum of 36% in the immediate postintervention phase and, subsequently, declined to range between 13% and 27%. This likely reflected operator challenges with using the length board equipment that was often described by PICU staff as being cumbersome to use and patient-specific characteristics such as contractures and/or deformities. The latter is especially important to consider as several PICUs in the United States increasingly provide care for children with multiple preexisting congenital and/or acquired comorbidities that affect linear growth.^17,18^ Alternative equation-derived length measurements from segmental measures of ulnar length or knee-heel length may be helpful in such circumstances as surrogates for measured stature.^19,20^

Although recently published guidelines recommend having a dedicated PICU NST to improve nutritional status assessment and delivery of nutrition,^21^ few studies have examined the role of a dedicated PICU NST to improve the performance of anthropometry measurements in the PICU.^22^ Similar to the results reported by Valla et al.^22^ in France, we noted that the PICU NST was effective in increasing provider awareness and education regarding nutritional status assessment. Our PICU RDs are present on daily bedside PICU rounds during weekdays to enhance nutrition awareness, knowledge among the clinical staff, and individualize recommendations and prescriptions to meet patient needs. As an important component of our project, the PICU NST provided “just-in-time” training for anthropometry measurements to bedside RNs. This training technique has been previously described to enhance performance and improve retention in a variety of procedural settings such as airway management, cardiopulmonary resuscitation, lumbar puncture, and central venous catheter placement.^23–26^ This methodology has also been utilized to enhance nursing and resident education and system-wide QI education to improve retention and engagement.^27–30^ The use of this methodology in our project may have contributed to the performance improvements with anthropometry measurements in the PICU.

The limitations of our QI project include lack of data on accuracy of measurements as the project focused primarily on completion of measurements. We attempted to overcome this limitation by both online education as well as “just-in-time” training methods on how to obtain such measurements in the correct manner. The results from this single-center project may not be generalizable to PICUs in other institutions. It is also possible that some of the improvements may have been confounded in the postintervention period by the Hawthorne effect. However, the same RDs collected data in both preintervention and postintervention periods during regular PICU bedside rounds to maintain consistency and minimize observer bias. Additionally, due to typical staff turnover and rotating clinical providers during the period of this QI project, it is possible that we were unable to ensure targeted educational efforts toward all providers. Finally, we were unable to study the direct impact of improvements in anthropometry measurements on key clinical and functional outcomes in the PICU such as mortality and length of stay due to confounding by simultaneous QI interventions implemented during the same time period in other clinical processes of care in the PICU.

Although our project demonstrated quantifiable improvements in performance of anthropometry measurements, significant gaps remained. Over a quarter of all patients did not have PICU admission weight measurements completed following institution of this project. Although it is likely that weight measurements may have been completed before PICU admission and therefore not repeated, it also reflects competing priorities for patient care and lack of sufficient personnel in the immediate period after the admission of a critically ill child. In turn, providers fail to recognize the potential for possible errors in such measurements obtained before PICU admission. Another important gap identified by the follow-up survey demonstrated differences in perceptions between ordering providers and bedside nurses with regard to lack of personnel and lack of importance of anthropometry measurements, suggesting the need for greater education and alignment of expectations across roles. With the incremental increase in RN FTEs required for anthropometry measurements, alternative strategies such as the use of nurse extenders or PICU technicians to assist in obtaining anthropometry measurements should be explored to reduce nursing burden and improve performance in these domains. The use of real-time data applications and metrics readily accessible to both ordering providers and bedside nurses may also heighten awareness of the gaps in anthropometry measurements for clinical care across roles and in turn help focus attention to improve measurement performance by aligning expectations.

Challenges to sustaining change in this project included loss of initial momentum and redirection of priorities. Additional hurdles were posed by difficulties with equipment location and maintenance as well as perceived lack of direct outcome benefits from anthropometry measurements. Solutions and strategies that are being examined by the PICU NST to sustain change and improve anthropometry measurements include ongoing education, establishing commitment to this QI effort as essence of culture, deriving more direct outcomes data (such as frequency of medication dosing errors related to weight/stature measurement errors), and continuous performance evaluation and individualized feedback to PICU staff.

## CONCLUSIONS

The process of obtaining anthropometry measurements in the PICU can be improved through the implementation of targeted QI methods by a dedicated PICU NST, but challenges exist to sustain these improvements over time due to competing clinical priorities.

## ACKNOWLEDGMENTS

We gratefully acknowledge the support of the PICU staff and patients with completion of this project.

## DISCLOSURE

The authors have no financial interest to declare in relation to the content of this article.
